# Resolving hidden pixels beyond the resolution limit of projection imaging by square aperture

**DOI:** 10.1038/s41598-023-30516-z

**Published:** 2023-03-01

**Authors:** Kelvin J. Xu, Gu Xu

**Affiliations:** 1 Fu Foundation School of Engineering and Applied Science, Columbia University, New York, NY 10027 USA; 2 Materials Science and Engineering, McMaster University, Hamilton, ON L8S4L7 Canada

**Keywords:** Biophysics, Cancer, Cell biology, Soft materials, Computational biology and bioinformatics

## Abstract

Projection imaging has been employed widely in many areas, such as x-ray radiography, due to its penetration power and ballistic geometry of their paths. However, its resolution limit remains a major challenge, caused by the conflict of source intensity and source size associated with image blurriness. A simple yet robust scheme has been proposed here to solve the problem. An unconventional square aperture, rather than the usual circular beam, is constructed, which allows for the straightforward deciphering of a blurred spot, to unravel hundreds originally hidden pixels. With numerical verification and experimental demonstration, our proposal is expected to benefit multiple disciplines, not limited to x-ray imaging.

## Introduction

Projection imaging by x-ray or others has been used extensively in radiography for medical diagnosis and structural examinations, because of its penetration power through the opaque human body and nontransparent packages^1–8^. However, the image resolution has always been limited by the size of the light source, usually in the sub-millimeter ranges^9–11^, being too coarse for biological cell identifications, which typically requires a resolution of micrometers^12,13^. This is simply caused by the dilemma that, the smallest pixel attainable, is correlated to the finite size of the light source. The latter cannot be too small, or the intensity becomes too weak to perform the diagnosis, within a limited time window for a patient to stay still, in the cases of chest x-ray, mammogram, or even computed tomography (CT)^9,14^.

**Figure 1:**
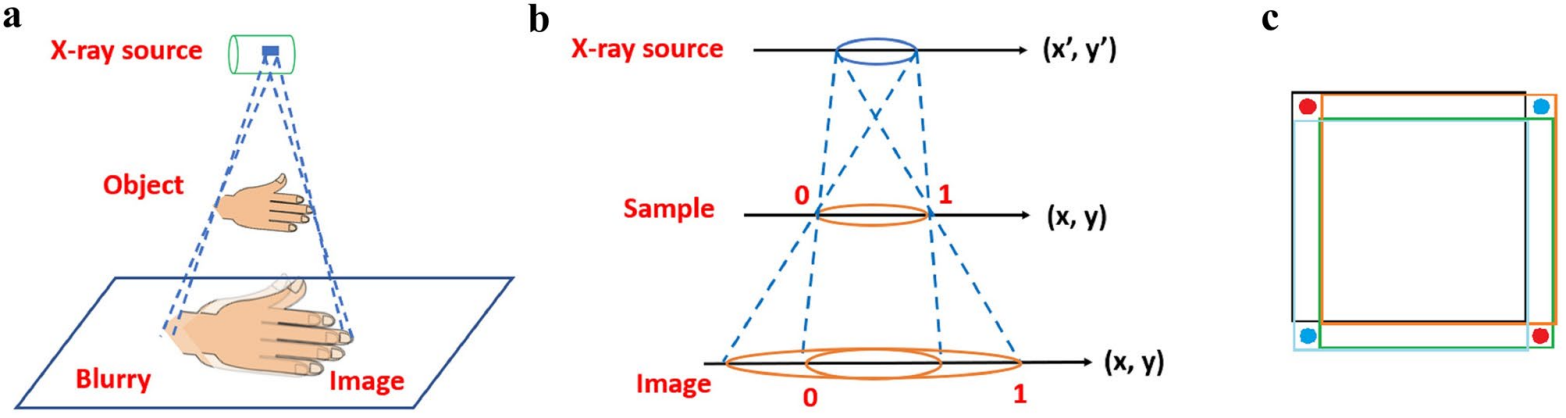
(**a**) Due to the ballistic nature of short wavelength x-rays, and submicron coherent lengths, a non-point source produces many projected beams through an object, all of which are then superimposed onto each other, to form a blurred image on the detector screen. (**b**) From the projection geometry, the image p(x,y) is simply given by the sample f(x,y) convoluted over the light source distribution g(x’,y’), where the scale of (x,y) for f, and p, can be adjusted to simplify the derivation. (**c**) The “square aperture difference” of p(x,y), convoluted by a point f with a square g, where the green square = p(x,y), orange = p(x,y + d), black = p(x + d,y + d), blue = p(x + d,y). To be consistent with the data matrix, the upper left corner is taken as the origin of (x, y), and d the amount of shift along the x,y axes. The resulting 4 p’s cancel each other everywhere, except for the positive red dots, sized by d, and negative blue dots, which will be dropped when re-written into an image.

The origin of this problem can be illustrated by the projected beam through ballistic geometry construction^15,16^, since x-ray has sub-nanometer wavelengths, and coherent lengths of less than a micron. As shown in Fig. 1a, an x-ray emitter, e.g. tube anode, can be considered as a collection of point sources. While each point acts as an origin of their respectively projected beams through the sample down to the detector, all of them are then superimposed onto each other, to form a blurred image on the detector screen^17–22^. Although, in principle, this blurriness could be resolved through the so called “point spread function” (PSF) of the x-ray source, via Fourier transforms and inversion, it has been rather impractical, if not entirely impossible, as the division of the PSF transform, usually of an oscillation function involving many zeros, ruins the operation.

Other possible remedies include miniaturized x-ray source, which inevitably reduces the flux^23,24^ and raises the exposure time. Though this is viable for materials studies^25^, it is unsuitable for patient diagnosis. Likewise, x-ray microscopy based on zone plates^26–30^ and coherent diffraction imaging^31–38^, despite their sub-micrometer resolution, rely on the much weaker diffraction which also requires inversion, thus unsuitable for the medical applications.

It is therefore the purpose of this report to solve this long standing problem since the advent of the radiography, by a combined method of hardware change and image computation, of resolving hundreds of pixels hidden within one blurred spot sized by the light source. Rather than the conventional circular cross-sectioned source, a square aperture beam is substituted, which leads to straightforward deciphering. With numerical verification and experimental demonstration, the strategy is readily extendible into the nano realm, as long as a much shorter wavelength is employed to maintain ballistic path geometry, to allow for a wide variety of imaging applications.

## Proposed methodology

From the projection geometry, Fig. 1b, the image **p** is a sample **f** convoluted over the source **g**, where the scale of (x,y) for **f**, and **p**, can be adjusted to simplify the derivation:

1 $${\text{p}}\left( {{\text{x}},{\text{y}}} \right) = \iint {{\text{f}}\left( {{\text{x}} + {\text{x}}^{\prime } ,{\text{y}} + {\text{y}}^{\prime } } \right){\text{g}}\left( {{\text{x}}^{\prime } ,{\text{y}}^{\prime } } \right){\text{dx}}^{\prime } {\text{dy}}^{\prime } }$$

For a square light source **g**, consider a pinhole sample, where **f** is a δ-function, thus **p** becomes the same as **g**, a square instead of a pinhole. If we construct the “square aperture difference”: p(x,y)−p(x,y + d) + p(x + d,y + d)−p(x + d,y), i.e., the original square image, minus the horizontally and vertically shifted images, plus the image shifted in both directions, the 4 squares cancel each other everywhere except at the corners, as shown in Fig. 1c, labelled by the positive red dots and negative blue dots, and sized by d, the amount of shift. As the digital image takes only the positive contribution, we have achieved deciphering of a well separated twin dot image of the original pinhole, from the blurred square image.

For the pinhole sample **f**, and a square light source **g** of size D, the resulting square image **p** is in the same order of magnitude as D. As this image square is the smallest spot obtainable by the detector, the image resolution is limited to the size of light source. Therefore, the minimum detector pixel size, is in the same order of magnitude of D, and it would be a waste if the detector pixel size <  < D. However, if a much finer detector is employed, after the square-aperture deconvolution outlined here, the resolution is much enhanced to become the shift amount, d, which is <  < D, the source size. Thus hundreds of pixels are now resolved from one originally blurred. In general, any sample can be considered as the superposition of many pinholes. Therefore, the same strategy can be used to decipher a general image, to obtain a much finer resolution determined by the shift d, than the original D size of the light source.

**Figure 2:**
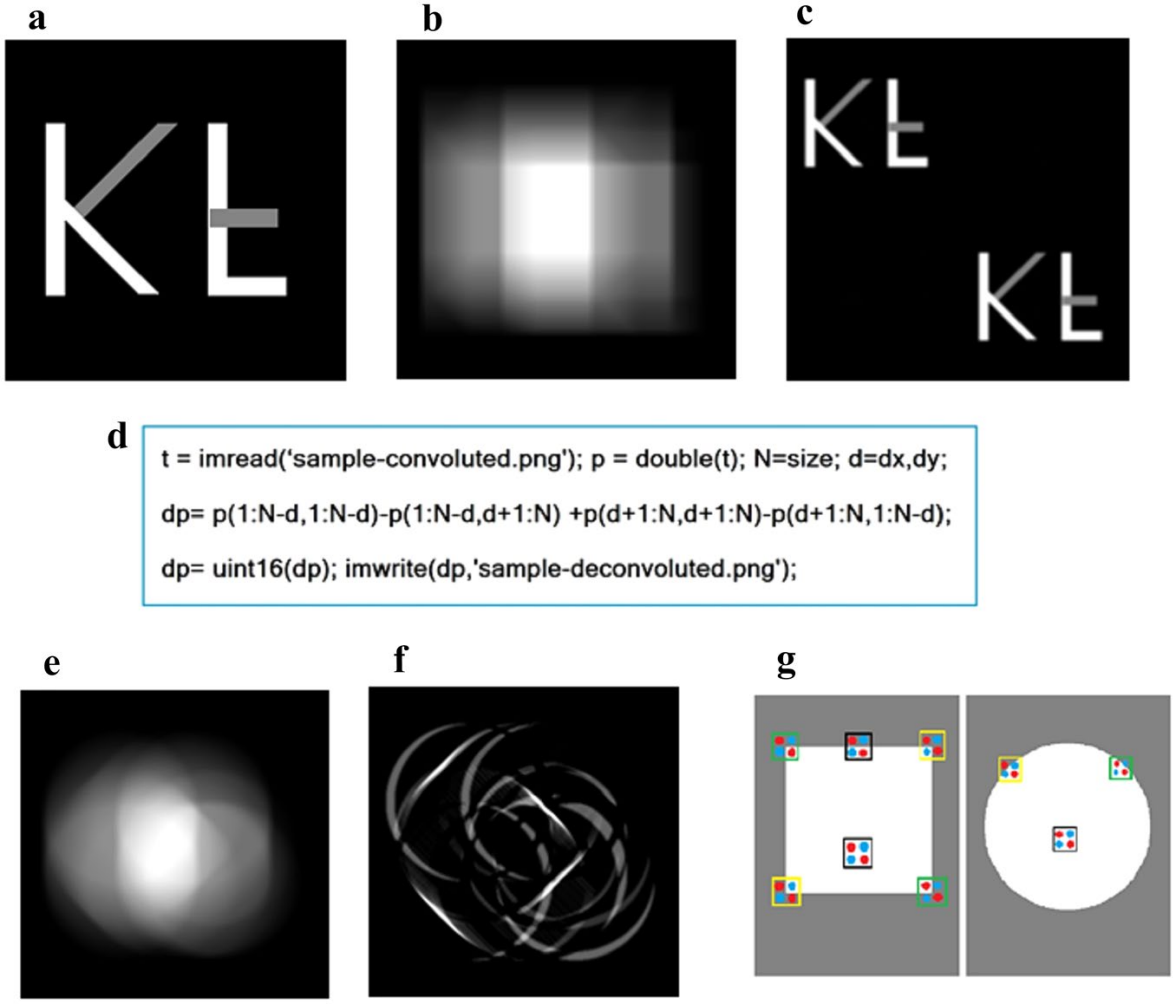
(**a**) Numerical construction of an f(x,y) of “K” and inverted “F”, with 0, 0.5 and 1.0 intensities, over a matrix of 200 × 200, to serve as a demo sample. (**b**) f convoluted via Eq. (1) by a square source g of 200 × 200, the resulting p(x,y) are blurred, featuring square shaped spots caused by square light source. (**c**) Decipher matrix p(x,y) by the “square aperture difference” producing a twin image, separated by the source size. (**d**) Matlab™ commands for image-data, and deciphering by “square aperture difference”, (**e**) For the usual circular beam source, the convolution of 2a becomes very different; (**f**) The de-convolution of 2e produces a collection of curved lines, the letters are invisible; (**g**) Why the usual circular beam fails: for a pinhole f, the circular shape g forms a ring shaped rising edge, giving non-zero contribution along the circle, whereas the square g makes zero contribution from the edges, as the red and blue dots cancel completely.

To expand this from the pinhole f, a sample of “K” and inverted “F” constructed numerically, with 0, 0.5 and 1.0 intensities, as shown in Fig. 2a, is convoluted by a square of 200 × 200, to mimic the projection imaging by a square light source, Fig. 2b. The resulting p(x,y) are blurred, with minimum features resembling square shapes caused by square light source. After importing the image to a matrix p(x,y), apply the “square aperture difference” of p, to obtain the recovered twin image of the letters, Fig. 2c. This is done by calculating the difference of the two diagonal sums for a square element, with a single line of command in Matlab™, of adding and subtracting the same image data matrix, after shifting horizontally or vertically over d, Fig. 2d. The same can be accomplished by a double calculation loop, going through the addition and subtraction of all the million pixels of the four ‘shifted’ pictures, which increases computational complexity, but ends up with a similar running time of less than a few seconds on a usual PC. The resulting twin images are separated by the diagonal of the source square, enough to resolve the original blurriness. It can be found that, the resulting picture resolution d is related to (source size D)/(signal-to-noise ratio, SNR), as the signal slopes down from the center over D.

Such a scheme would not work, however, for the usual circular light source. As one can see from the convolution shown by Fig. 2e, and the deconvolution shown by Fig. 2f, the result is just a collection of curved lines. This can be understood by imagining a “pinhole sample”, where the circular light source forms after the cancellation a ring shaped bright edge, leading to the non-zero contribution along the circle, whereas the square source makes zero contribution from the edges after the cancellation, except for the corners, Fig. 2g.

## Results and discussion

**Figure 3:**
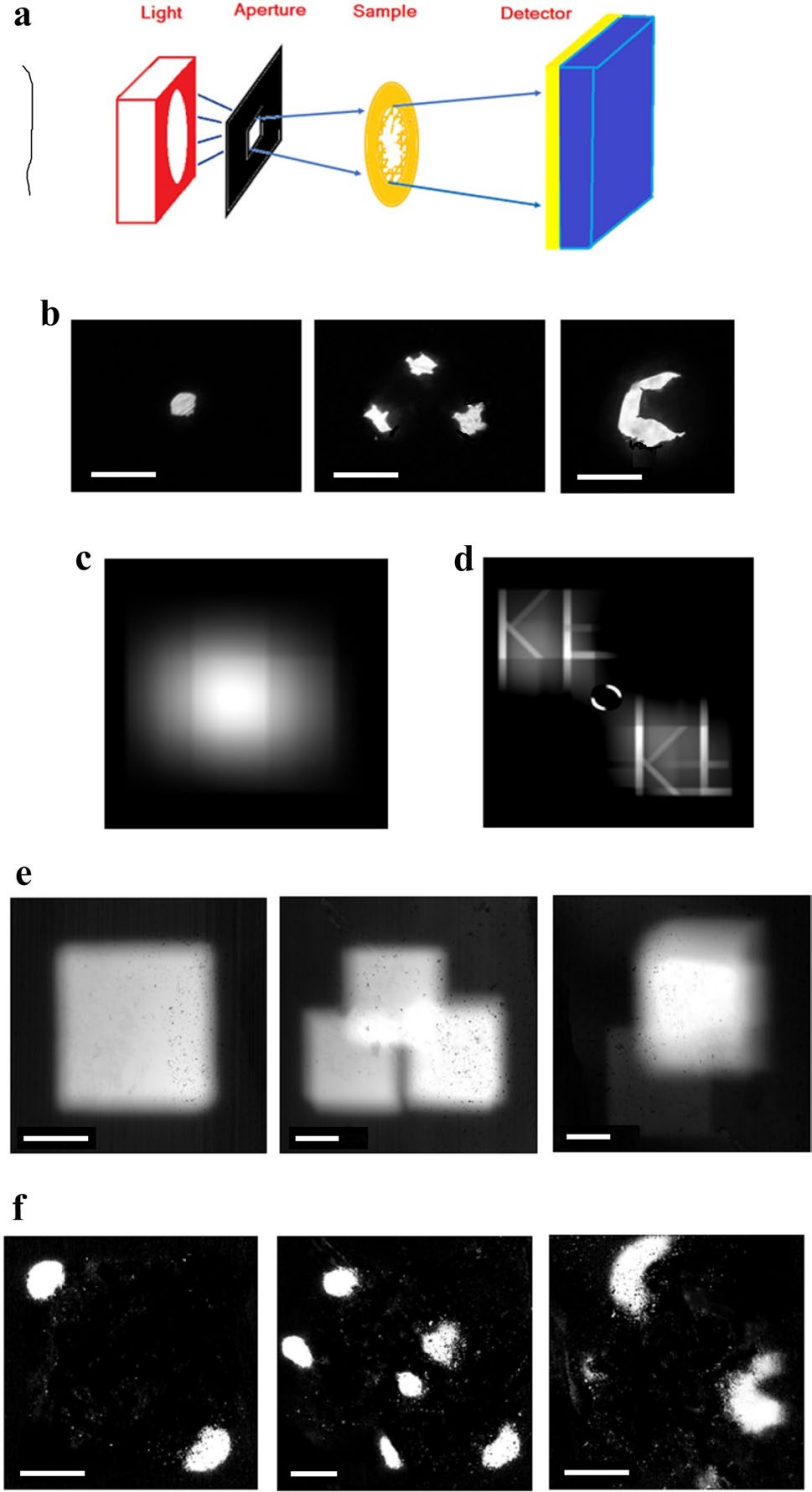
(**a**) The experimental setup of a non-point light source of wavelengths centered around 550 nm, square aperture by 2 perpendicular slits of 1 mm separation via thin metal strips, sample by a pinhole punched metal foil, and 3 × 5mm^2^ image sensor from a digital camera, with the lens assembly removed. The aperture-detector distance is about 50 mm, with the sample placed in between. (**b**) microscope photos of the samples made of metal foil; one pinhole on the left; triple pinholes in the middle, and the letter “C” to the right. Scale bar length: 300 µm. (**c**) For a common non-point light source, the intensity may not be uniformly distributed across the square aperture. Thus Fig. 2b was recalculated by incorporating a Gaussian decaying factor of exp(− R^2^/R_o_^2^) on top of a square g(x’,y’), where R is the radius from the center, and R_o_ is about 70% of the half width of g. (**d**) Despite the resulting p(x,y) starts to show some artifacts, the de-convolution of 3c is similar to Fig. 2c, due to the fact that it is slowly-varying, when compared with the letters. (**e**) The resulting photos from the metal foils in 3b, taken by a CMOS sensor of digital camera: on the left, almost a perfect square like spot is produced by the light source through a single pinhole in 3b; in the middle, three overlapping squares are formed by the triple pinholes in 3b; to the right, the “C” in 3b leads to a square-like bright spot. Scale bar length: 400 µm. Note that these squares are the smallest spots attainable due to the finite sized light source, where the original pinholes and letter “C” are blurred. (**f**) The de-convolution results of 3e by Matlab™ command in 2d: on the left, the “square” produces after the “square aperture difference” a pair of bright dots, corresponding to the two red dots in 1c; in the middle, the triple pinhole forms two sets of triplets, with one on the upper left and the other on the lower right; to the right, the doubled “C”s are evident, despite the noise. Scale bar length: 400 µm. Compare with 3e, the resolution is much improved and the blurred images are now resolved.

As commercial x-ray tubes are not easily manipulated, and micron pixel detectors are yet to be available for x-rays, experimental demonstration can be done using visible light from a common LED lamp, and CMOS of digital cameras^39^, with (1) a square aperture formed by arranging two perpendicular slits of thin metal strips, and (2) a CMOS image sensor from a digital camera with the lens removed, plus (3) a pinhole punched metal foil to serve as a ‘sample’, Fig. 3a,b. Since the intensity might not be uniform across the square aperture for a common light source, in order to confirm the de-convolution method, Fig. 2b was recalculated by incorporating a Gaussian decaying factor of exp(-R^2^/R_o_^2^) on top of the square g, where R is the radius from the center, and R_o_ was set at about 70% of the half width of g, Fig. 3c. The de-convolution result of 3c is very similar to that of 2c, due to the fact that it is slowly-varying, when compared with the letters, shown by Fig. 3d.

Single shots of the digital camera produced JPG images, Fig. 3e. On the left, a square shaped spot is produced by the light source through a single pinhole (sample in Fig. 3b, left). Notice that the smallest spot size (resolution) in the pictures of Fig. 3e, is determined by the light source shape and size, viz., a square of about 1 × 1 mm^2^, defined primarily by the double slits of the light source, and adjustable through the sample-detector distance. After the de-convolution, we obtained the “square aperture difference” of the original image, horizontally shifted image, vertically shifted image, and the image shifted in both directions, as shown in Fig. 3f. Here, however, the smallest spot size was much reduced, or the resolution much improved. For example, on the left of Fig. 3e, the 1 mm “square” produced after the “square aperture difference” a pair of diagonal bright spots, corresponding to the two red dots in Fig. 1c, which were about an order of magnitude less in size, when compared with the original square. Therefore, we achieved resolving almost a hundred pixels hidden in the original square spot. The pixel number can be increased if a smaller d, the shift amount, is adopted, when over hundred pixels can be resolved out of one original square spot of size D.

When more pinholes are found in the sample, as shown by the middle panels of Fig. 3b, the same analysis can be applied, where the triple pinholes produced three overlapped square like spots in the middle panel of Fig. 3e. Again, the resolution here was limited by the light source size of millimeter square. However, the spot size was much reduced after the de-convolution by “square aperture difference”, as shown in the middle of Fig. 3f. As discussed above, in the middle panel of Fig. 3e, the resolution is determined by the square of the lamp size, when 3 pinholes were blurred to become three overlapped squares, or three circular spots if we use circular cross-sectioned source. Obviously, it seems to be a waste to employ fine pixel detectors. However, when we de-convolute the squares from the fine pixel detector by “square aperture difference”, three well separated pinholes are resolved, leading to a much finer resolution in the middle panel of Fig. 3f.

While the same analysis can be extended to more complicated samples, such as the letter “C” in the right panel of Fig. 3b, the shift amount, d, which is also the de-convoluted image resolution, depends on the source size (D)/(SNR). Although the precision of the square aperture, etc., was limited by the cutting tools, which may have led to the distortions of the recovered pinholes, the main features and the sample letter “C” were clearly visible, shown in the right panel of Fig. 3f. Also, the orientation and angles of the square aperture were found to be quite tolerable to deviations. In addition, the quality should be much improved if PNG type of photos are adopted, which possess 2^16^ grey scales, instead of the usual 2^8^ for JPG format, which poses a major limitation here.

Compared with point source projection imaging, when the signal may become too weak and swamped by noise, the convoluted pattern of the finite-sized source is easily surviving, due to the much enhanced signal by then. On the other hand, although this may be grouped with the Coded Aperture^40–42^, or other super-resolution radar imaging technique^43,44^, there is a fundamental difference here: whereas the Coded Aperture talks mainly about the patterned grid covering the entire aperture, such as Fresnel Zone Plate^26–30^, the proposal here is about changing the Boundary/shape. For example, in this experimental demonstration, the quantified resolution obtainable by the 1 mm cross square aperture is about 0.1 mm, which was mainly limited by the square edge roughness, whereas the smallest ‘pixel’ of a similarly sized circular aperture would be about 1 mm, due to the projection overlapping discussed above.

## Summary

We have found a solution to the problem of resolution limit due to the source intensity, and achieved a robust scheme of unraveling hundreds of pixels hidden within an originally blurred spot due to a finite source size. Rather than the conventional circular shaped beam, a square cross-sectioned beam proves to be an easy solution. Due to the simplicity of the de-convolution, the strategy can be extended to a micrometer source to achieve nanometer resolution for bio-samples, as long as a shorter wavelength can be employed to maintain the ballistic path geometry, to ignore the diffraction. Since the hard x-ray wavelength is about 1/5000 of that of the visible light, the pinhole sample of 0.1 mm is equivalent to a size of 20 nm in the case of x-ray. Therefore, the experimental demonstration here shows the potential of nanometer resolution, should the corresponding x-ray source and detector become available. As well as the high precision square apertures can be constructed by the available nano-fabrication today, whose edge roughness determines ultimately the finest pixel, or the resolution of the proposed method here. The easy implementation is expected to offer a sweeping advancement, to not only chest x-ray, mammograms, but also 3D CT scans, which are all based on 2D projections, as well as the development of nanometer pixel detectors.

## Data Availability

The datasets used and/or analysed during the current study available from the corresponding author on reasonable request.
